# Self-rating via video communication in children with disability – a feasibility study

**DOI:** 10.3389/fpsyg.2023.1130675

**Published:** 2023-05-02

**Authors:** Magnus Ivarsson, Anna Karin Andersson, Lena Almqvist

**Affiliations:** ^1^Department of Behavioural Sciences and Learning, Linköping University, Linköping, Sweden; ^2^Division of Physiotherapy, School of Health, Care and Social Welfare, Mälardalen University, Västerås, Sweden; ^3^CHILD, Jönköping University, Jönköping, Sweden; ^4^Division of Psychology, School of Health, Care and Social Welfare, Mälardalen University, Västerås, Sweden

**Keywords:** cognitive accessibility, developmental disability, interview, NDD, Picture My Participation, participation, Talking Mats, video application

## Abstract

**Background:**

Different barriers may hinder children with developmental disabilities (DD) from having a voice in research and clinical interventions concerning fundamentally subjective phenomena, such as participation. It is not well-investigated if video communication tools have the potential to reduce these barriers.

**Aim:**

This study investigated the feasibility of administering a self-rating instrument measuring participation, Picture My Participation (PmP), via a video communication tool (Zoom), to children with DD.

**Materials and methods:**

PmP was administered to 17 children with DD (mean age 13 years). The pictorial representations of activities and response options in PmP were displayed in a shared PowerPoint presentation, enabling nonverbal responses with the annotate function in Zoom. Child and interviewer perceptions of the interview were measured through questionnaires developed for the purpose.

**Results:**

All the children completed the interview. Most PmP questions were answered, and no adverse events were registered. Technical issues could generally be solved. No special training or expensive equipment was needed for the interviews.

**Conclusion:**

Interviewer-guided self-ratings of participation and related constructs through video communication may be a feasible procedure to use with children with DD from age 11.

**Significance:**

Offering video communication may increase children’s chances to contribute subjective experiences in research and clinical practice.

## Introduction

Children with developmental disabilities (DD) face different barriers hindering them from having their voices heard in in-real-life (IRL) interviews in both research and clinical practice (Varghese et al., 2015; Adugna et al., 2020; Doherty et al., 2020). Video communication-based procedures have an intuitive appeal as a way of reducing some of these barriers and thereby increasing participation. However, examining the feasibility of such procedures before applying them on a larger scale is important. The current study aims at exploring the feasibility of administering a self-rating instrument via a video communication tool to children with DD.

In the present study, the term DD refers to a set of conditions characterized by persistent physical and/or mental impairments affecting multiple major life activity areas, with an onset during the developmental period (in line with the Developmental Disabilities Assistance and Bill of Rights Act of 2000, 2000). Beyond the neurodevelopmental disorders listed separately in the International Classification of Diseases and Related Health Problems (11th ed.; ICD-11; World Health Organization, 2022), such as disorders of intellectual development or developmental language disorder, the term DD also tends to include diagnoses from other parts of the ICD-11 such as cerebral palsy and spina bifida. Language impairments are common in DD, sometimes as a characteristic feature (e.g., pragmatic language impairments in autism spectrum disorder; World Health Organization, 2022), and in other cases as a condition co-occurring with another disability, such as attention deficit hyperactivity disorder (Mueller and Tomblin, 2012), autism spectrum disorder (Kjellmer et al., 2018), and cerebral palsy (Mei et al., 2016). Accordingly, in a sample of children with different DD, it would be reasonable to expect impairments in different aspects of communication, including pragmatic, receptive, and expressive language.

There are strong reasons for including the perspective of the child in research and health services targeting important everyday life aspects, such as participation and mental health (Nilsson et al., 2015). By using the term children in this study we refer to people 0 to 18 years of age. A fundamental ethical principle reflected by article 12 in the United Nations Convention on the Rights of the Child (1989), states that all children capable of forming views should be assured the right to express those views in matters affecting them. There is also a methodological argument to be made. Few studies show acceptable agreement between child and parent or teacher ratings (Achenbach et al., 1987; De Los Reyes et al., 2015; Huus et al., 2015; Dada et al., 2020), indicating that the omission of the child’s subjective experience could lead to an incomplete understanding of the phenomena studied. Still, the subjective perceptions and experiences of children with DD are often neglected (see for example van Steensel et al., 2011; Downs et al., 2018).

One possible reason why the voices of these children are often excluded in this field of research is that many children with DD entail deficits in the cognitive and communicative abilities involved in self-assessment and self-rating (Beddow, 2012; Fujiura and RRTC Expert Panel on Health Measurement, 2012). For example, self-rating scales, in general, presuppose that the respondent can comprehend a certain level of written or spoken language and produce a verbal or manual response, i.e., abilities that may be impaired in developmental language disorder and many other DD. However, in the last decades, attention has shifted from the abilities of the respondent child to the properties of the materials and procedures used in assessment (Döring, 2010; Saywitz and Camparo, 2014). A cognitively accessible design (in self-rating questionnaires) anticipates respondent variability in cognitive abilities and reduces cognitive demands, i.e., the specific mental functions that a questionnaire implicitly assumes in a respondent (Kramer and Schwartz, 2017). It may also help respondents to interpret and respond to assessment items as intended. Thus, the accessibility of a self-rating questionnaire is related not only to the objective accessibility of the questionnaire but also to how the respondent perceives it (Maxwell et al., 2012).

This shift in focus, from body functions to contextual factors (World Health Organization, 2001), is demonstrated by the innovative attempts to support the self-rating of subjective experiences in children with DD through the use of different assistive aids, such as pictures or symbols (see for example Gullone et al., 1996; Scott et al., 2011; Boström et al., 2016; Arvidsson et al., 2021). The scale applied in the present study, Picture My Participation (PmP), is one example of a scale intended to be accessible to children with different levels of cognitive and communicative functioning. By using visual support and a relatively flexible procedure (within defined limits), PmP measures core aspects of participation (attendance and involvement) in everyday activities in children (Arvidsson et al., 2020, 2021). As such, PmP resonates with the definition of participation in the Family of Participation-Related Constructs (fPRC) framework, which identifies attendance (i.e., being there) and involvement (i.e., the experience of participation while attending) as the two essential components of participation (Imms et al., 2016, 2017).

However, cognitively and communicatively inaccessible materials and procedures are not the only factors hindering children with DD from participating in scientific studies. The COVID-19 pandemic has been the most recent example of a hinder to IRL interviews but other factors relating to the child or family, e.g., physical inaccessibility, lack of transportation, and lack of privacy, may also cause difficulty in including children in research and may hinder necessary health care interventions (Varghese et al., 2015; Adugna et al., 2020; Doherty et al., 2020). Such obstacles may have an impact on decisions on whether or not to include the children themselves in the assessment of mental health and participation-related constructs or to settle for proxy ratings.

For this reason, interviewing or data collection over a physical distance seems like an appealing solution. Video communication could have some potential advantages over the telephone, SMS, or chat interviewing since it allows the combination of spoken language with forms of augmented and alternative communication (e.g., body language, sign language), which may be necessary for successful communication with some children with DD (Kaiser et al., 2001; Stephenson and Limbrick, 2015). At the same time, the video communication tool applied, or the video format *per se* may contain cognitive and communicative barriers restricting participation in interviews for the same children. It is largely unknown how this change in the procedure may impact cognitive and communicative accessibility when collecting self-reported data on subjective phenomena such as participation. The video format and the associated digital environment may involve both elements that increase and decrease cognitive and communicative demands. Assistive aids, such as pictures or symbols, could be difficult to transfer to the digital environment. They are often reliant on IRL interviewing and can thus be dependent on situational and geographical conditions (Kramer et al., 2009). At the same time, it is also possible that the digital environment could enable new forms and uses of pictorial support. The direction of this effect may also be dependent on child factors. For example, it may be easier to combine a digital environment with different response formats (touch screen, eye control, etc.), enabling children with different levels of motor and communication impairments to respond to questions in various ways. Whether the administration of self-rating instruments by video communication increases or decreases the accessibility of children with DD needs to be further investigated.

As a first step, there is a need to evaluate the feasibility of administering self-rating instruments via video communication to children with DD before implementing the procedure in larger-scale studies. A feasibility study such as this makes it possible to foresee barriers and minimize negative consequences in later stages (Tickle-Degnen, 2013). The study aimed to investigate the feasibility of administering a self-rating instrument, PmP in this case, via a video communication tool (Zoom) for children with DD, including the subjective experiences of children and researchers in the process. By doing so, aspects of the children’s participation (i.e., attendance and engagement) in the digital environment while being interviewed were identified and discussed.

## Materials and methods

Based on a summary of the literature, Orsmond and Cohn (2015) have identified five main objectives for a feasibility study: (1) recruitment and sample characteristics, (2) procedures and measures, (3) study acceptability, (4) resources and ability to manage study, and (5) preliminary evaluation of participant responses. We used these five objectives as a structure for evaluating the feasibility of using a video communication tool for guiding self-ratings of PmP with children with DD.

### Participants

**Figure 1:**
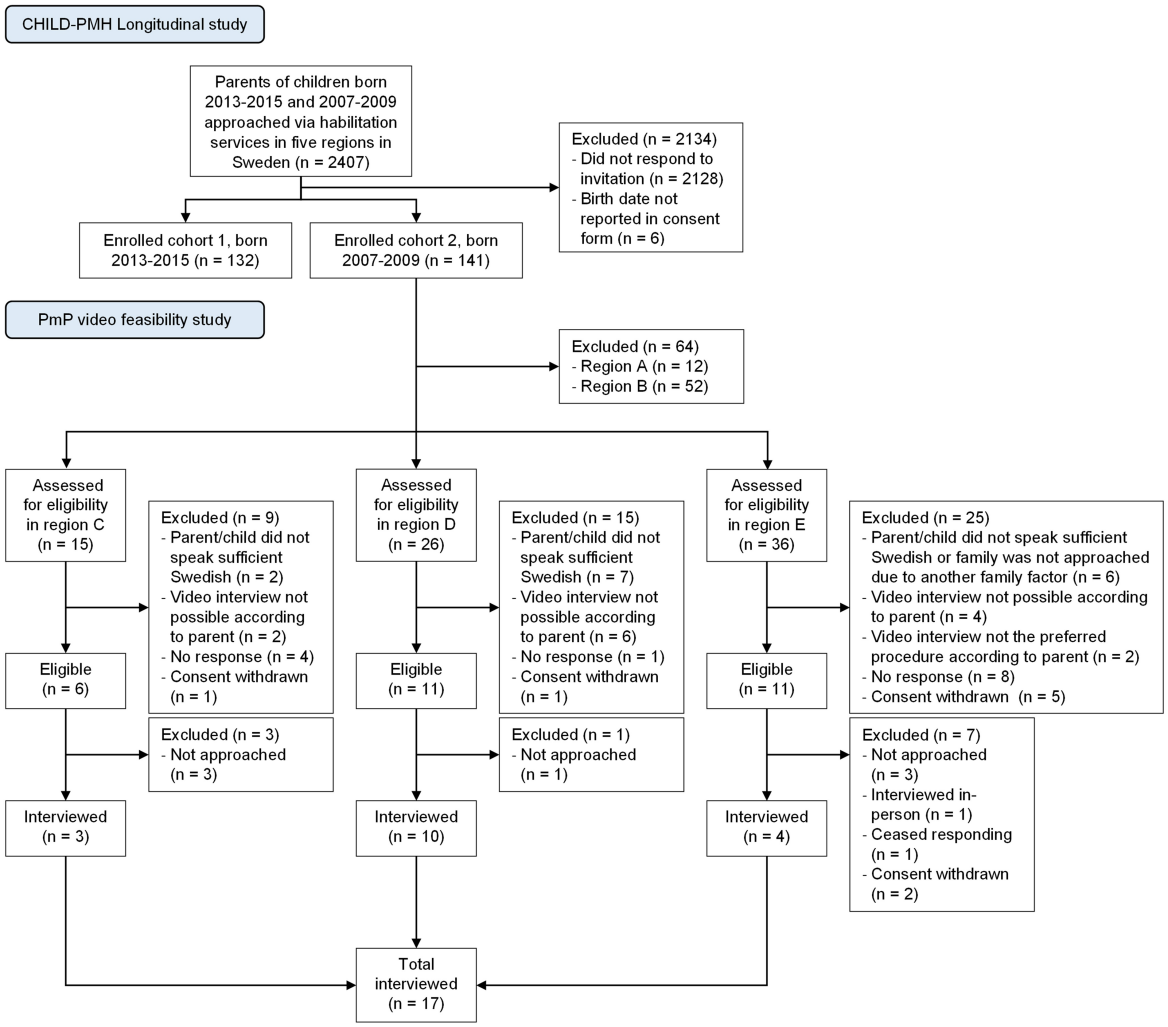
Recruitment strategy and flow of participants through the study.

Seventeen children with DD were recruited from the older cohort (born 2007–2009) of an ongoing longitudinal study of mental health and participation in children with DD in Sweden (CHILD-PMH). All families enlisted at the habilitation services in five regions in Sweden were invited to participate in CHILD-PMH via mail (see Figure 1 for a description of the flow of participants through the larger longitudinal study and the present study). The invitation mail was written in Swedish but contained information on how to access Arabic, English, or Somali translations. The habilitation services in Sweden serve children with DD who have substantial support needs, such as those with intellectual disability, autism (although this differs between regions), and cerebral palsy. Generally, they do not serve children with attention deficit hyperactivity disorder, developmental coordination disorder, specific learning disorders, etc. Children with the severest disabilities, e.g., children with substantial impairments in movement and intellectual functioning, are almost always enrolled in habilitation services. The level of enrollment varies more for children with milder levels of disability. For reasons of convenience, the sample of participants in the present study was drawn from three of the five participating regions in CHILD-PMH. During the initial contact with parents in the longitudinal study, they were asked if they believed that their child would be interested in participating in the current feasibility study and if data collection via video communication would be plausible for their child if adaptations were made. Exclusion criteria were (1) the parent not understanding the information about the study presented orally in plain Swedish or (2) the child having a type or degree of disability that would make it impossible to guide the child through the self-rating. Child consent was collected orally in connection with the interview. The CHILD-PMH project has been approved by the Swedish Ethical Review Authority (case number 2019-05028).

## Material

### Video-communication platform: Zoom technology

The choice of video communication platform was based on the following criteria: (a) using it was not associated with any costs for the child’s family, (b) it could be run in commonly used web browsers on different types of devices, (c) it had a function that enabled both the host and guests to draw and make notes in the same document within the interview without having to open additional applications, (d) it was well known to the average internet user, and (e) it was intuitive and fairly accessible in cognitive terms (e.g., users do not have to go through a lot of text before launching the software). Microsoft Teams and Zoom were both considered with these criteria, and Zoom was chosen based on the criterion of allowing annotations in both the application and the web-based versions.

### Picture My Participation

The self-rating instrument PmP (Arvidsson et al., 2020) is developed for children and youths aged from five to 21 years of age, to measure participation in 20 different home, community, and social activities. PmP is administered as a guided conversation, using pictures from the aided Picture Communication Symbols (Fuller and Lloyd, 1997) illustrating the items and the different possible replies (Willis et al., 2015), and yield quantitative data on aspects of participation. During the interview, when performed IRL, the interviewer and the child sit side-by-side at a desk, looking and talking about the material placed in front of them. PmP helps children to identify participation from four aspects: frequency of attendance of activity, level of involvement when performing an activity, choice of three important activities determined independently, and evaluation of perceived barriers to and facilitators of participation. The frequency of attendance is rated on a four-point Likert scale visualized by baskets filled with apples, where a full basket corresponds to ‘always,’ three apples in the basket corresponds to ‘sometimes,’ one apple corresponds to ‘seldom’ and an empty basket corresponds to ‘never.’ Perceived involvement is rated on a three-point Likert scale visualized by three pictures showing a very (actively) involved child, a child observing peers who are active in a play (less involved), and a child who is not at all involved, respectively. Consequently, PmP is designed so that children with no or very limited ability to produce spoken language can respond to most items.

#### Translation to the video communication environment

An essential aspect of PmP is the use of a Talking Mats approach (Cameron and Murphy, 2002) with visual representations of activities and response options. In transferring the approach to the digital environment, simplicity was prioritized over exact resemblance to the non-digital version of Talking Mats, since it was deemed important that the participants did not have to switch between multiple applications during the interview or download additional applications. For this reason, all pictures were inserted into PowerPoint slides and arranged in a Talking Mats manner, meaning two slides per activity, one with the response options relating to attendance (‘Never,’ ‘Not really,’ ‘Sometimes,’ or ‘Always’) aligned above the activity and one with the involvement response options (‘Not,’ ‘Somewhat,’ or ‘Very’). Examples of the attendance and involvement questions are displayed in Supplementary Figures S1, S2. One slide with 20 pictures representing all the activities was created for the part of the interview where the child has to choose the three most important activities. For the part concerned with barriers and facilitators, each activity was displayed on a separate slide with the barriers and facilitator template pictures aligned above it. Instead of having the children pick up, or drag, a picture and placing it under the favored response option, as when guiding children through self-ratings in PmP IRL, the children were instructed to mark the response of their choice using the annotate function within Zoom or if possible and if they preferred, they could just tell their response.

### Ten Question screen

Ten Question screen (TQS, Durkin et al., 1991, 1995) is a parent-report screening tool developed to detect childhood disabilities in low and middle-income countries. In 10 closed binary questions the child’s vision, hearing, movement, cognitive functions, and seizures are addressed. TQS was completed by the primary caregivers to describe the nature of their child’s disabilities, either by telephone interview or questionnaire.

### Registration form and feasibility questionnaire

To help the interviewer keep track of important aspects of feasibility during the interview, a short interviewer registration form was developed. The form included headings to note the duration of the interview, the number of breaks, technical disruptions, and adverse events.

**Figure 2:**
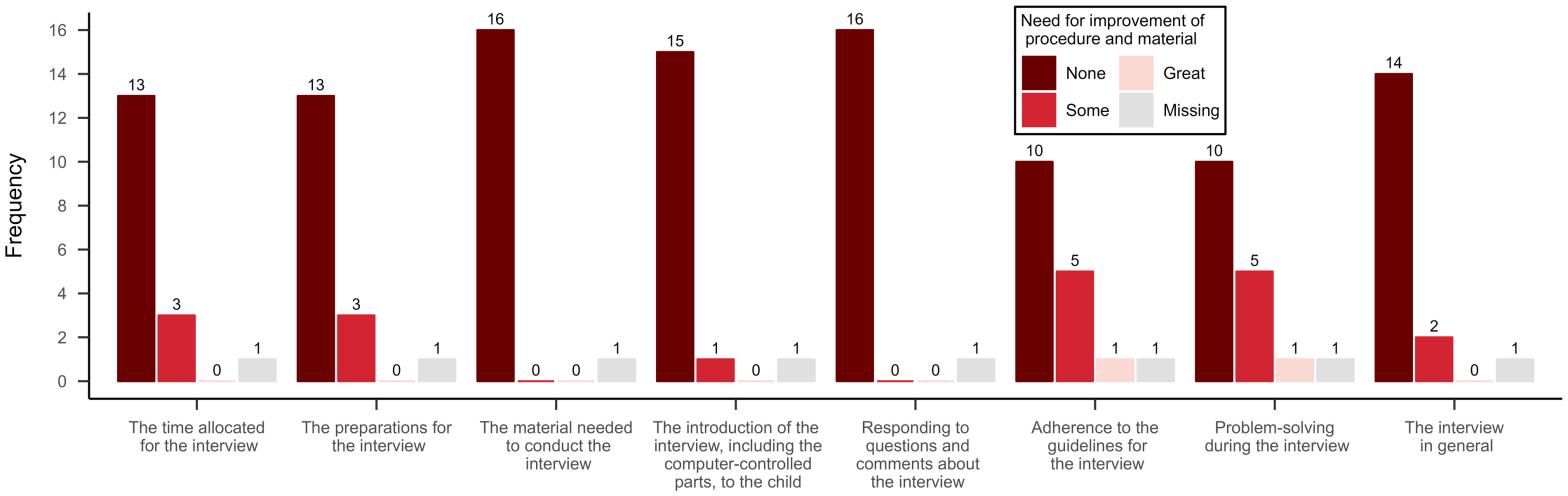
Interviewer rated need for improvement of procedure and material.

The interviewers’ perspective on the room for improvement of the material and procedure was measured with an eight-item questionnaire (see Figure 2), with a three-graded Likert-style scale (‘No room for improvement,’ ‘Some room for improvement,’ and ‘Great room for improvement’), developed by the research team. The interviewers responded to the questionnaire immediately after having finished an interview and were instructed to contemplate to which degree they could see room for improvement across the domains. The scale also contained an open question about any need for changes in procedure and content. Further, a simple logbook was developed to keep track of changes made between interviews. All described scales and forms were developed to fit the aims and questions proposed by Orsmond and Cohn (2015).

**Figure 3:**
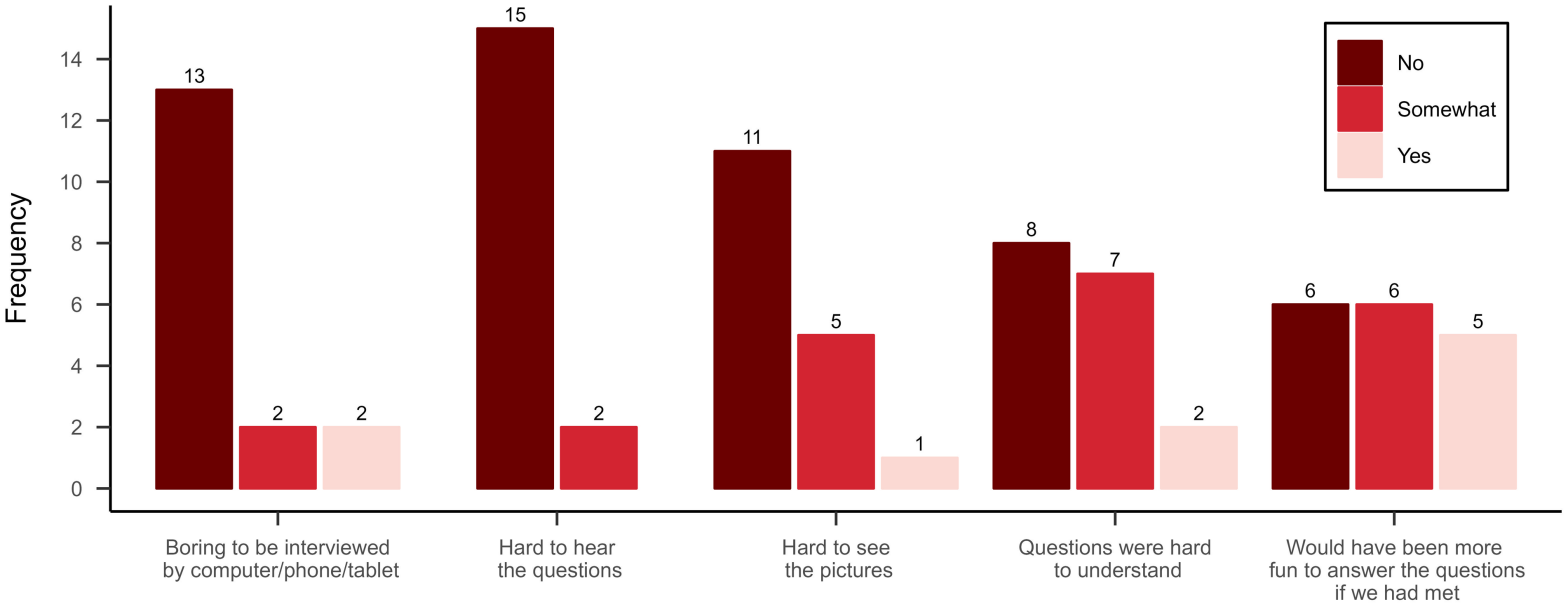
Participants attitudes toward the interview and the digital format.

To evaluate the children’s attitude toward the interview in general, and the digital environment in particular, a scale was developed including five items (see Figure 3) with a three-graded Likert-style response scale (‘Yes,’ ‘Partly,’ and ‘No’) and two open-ended questions (‘What would have been better/worse if I would have come to your home for the interview instead of conducting it via video?’ and ‘What can we improve if we are to interview more children via video in the future?’). The questionnaire was added to the same PowerPoint presentation as the PmP items, and the questions were displayed one at a time with response alternatives augmented with smiley-like faces in different colors.

### Procedure

A total of 28 of the 77 children assessed for eligibility met the inclusion criteria for the current study. Of these, 17 were asked to participate and agreed to a short 10–15-min preparatory meeting aiming to (a) test if the child was able to log on to a Zoom meeting, (b) if he/she could use the annotate function in Zoom to choose cards in a simple PowerPoint-based memory game, and (c) to collect informed consent to participate in the study directly from the child. The PmP interview was then scheduled at a separate time and day in all but one case. All interviews were conducted by the first author (a Ph.D. student and clinical psychologist with years of experience in interviewing adolescents with disabilities), the second author (a Ph.D. and physiotherapist with extensive clinical experience in interviewing children with disabilities), or a Ph.D. student working in the CHILD-PMH project. Parent participation in the interview was accepted but the interviewer made clear that it was the child’s own opinion that was the focus of the interview, and this instruction was repeated during the interview if necessary. The feasibility questionnaire was administered in direct connection with the PmP assessment, while the interviewer filled out the interviewer questionnaire immediately afterwards.

### Data analysis

The data were analyzed with descriptive statistics (i.e., counting of occurrences, and calculating means). All processing and analysis of data and visualizations were carried out in R (R Core Team, 2021) and RStudio (RStudio Team, 2020) with the *table1* (Rich, 2021), *ggplot2* (Wickham, 2016), and *patchwork* (Pedersen, 2020) packages (except Figure 1, which was made in Microsoft Word).

### Results

The findings are presented according to the five feasibility objectives suggested by Orsmond and Cohn (2015).

### Objective 1: evaluation of recruitment capability and resulting sample characteristics

The main question to ask to address this objective is: ‘Can we recruit appropriate participants?’ As expected, when recruiting participants from the habilitation services in Sweden where children with all levels and many different types of disabilities are enlisted, a substantial proportion of parents did not consider a video interview to be feasible for their child (see Figure 1). However, all children that were scheduled for an interview completed it according to plan, indicating that parents generally do not overrate their children’s abilities in this area. This could mean that a proportion (36.3% based on our preliminary findings) of children aged 11–14 years with DD may be eligible for a video-based version of the PmP interview. The eligibility rate may be higher, depending on the prerequisites of the assessment process. There is a chance that some of the excluded children would have managed the interview after all. This could certainly be the case when it comes to parents with another ethnic background, where the children sometimes speak Swedish more fluently than their parents. As in all research involving children with foreign backgrounds, some families were excluded because the parent did not understand the study information, which precluded them from providing informed consent.

Distinctive for the children participating in the interviews was that they tended to have more difficulty relating to movement (see TQS-5 in Table 1) and slightly less relating to cognitive (TQS-10) and communicative skills (TQS-8 and TQS-9) than the rest of the children in the CHILD-PMH cohort. Still, several of the children participating in the present study were rated to have some degree of difficulty with comprehension by their parents and the group was similar to the larger CHILD-PMH cohort in terms of age and average disability rate (mean across all TQS items).

**Table 1:** Characteristics of the participants in the current study and the rest of the children in the older cohort of the CHILD-PMH longitudinal study.

	CHILD-PMH (*N* = 68)	Current study (*N* = 17)
Gender		
Girl	23 (33.8%)	6 (35.3%)
Boy	45 (66.2%)	10 (58.8%)
Other	0 (0%)	1 (5.88%)
Birth year		
2007	20 (29.4%)	5 (29.4%)
2008	20 (29.4%)	7 (41.2%)
2009	28 (41.2%)	5 (29.4%)
Serious delay in sitting, standing, or walking (TQS-1)		
No	50 (74.6%)	12 (70.6%)
Yes	17 (25.4%)	5 (29.4%)
Missing	1 (1.5%)	0 (0%)
Difficulties seeing, either in the daytime or at night (TQS-2)		
No	59 (88.1%)	14 (82.4%)
Yes	8 (11.9%)	3 (17.6%)
Missing	1 (1.5%)	0 (0%)
Difficulties hearing (TQS-3)		
No	59 (88.1%)	15 (88.2%)
Yes	8 (11.9%)	2 (11.8%)
Missing	1 (1.5%)	0 (0%)
Child comprehends when parent asks the child to do something (TQS-4)		
No	8 (12.1%)	1 (6.25%)
Yes	58 (87.9%)	15 (93.8%)
Missing	2 (2.9%)	1 (5.9%)
Difficulty walking or moving arms or is weak or rigid in arms or legs (TQS-5)		
No	55 (82.1%)	9 (56.3%)
Yes	12 (17.9%)	7 (43.8%)
Missing	1 (1.5%)	1 (5.9%)
Sometimes has seizures becomes rigid or loses consciousness (TQS-6)		
No	56 (83.6%)	16 (94.1%)
Yes	11 (16.4%)	1 (5.88%)
Missing	1 (1.5%)	0 (0%)
Has learned to do things that other same-aged children do (TQS-7)		
No	31 (49.2%)	7 (41.2%)
Yes	32 (50.8%)	10 (58.8%)
Missing	5 (7.4%)	0 (0%)
Speak at all (TQS-8)		
No	10 (15.2%)	0 (0%)
Yes	56 (84.8%)	16 (100%)
Missing	2 (2.9%)	1 (5.9%)
Mentions at least one thing (TQS-9)		
No	9 (13.6%)	0 (0%)
Yes	57 (86.4%)	17 (100%)
Missing	2 (2.9%)	0 (0%)
Seems to have difficulty comprehending or is slow (TQS-10)		
No	24 (36.9%)	8 (47.1%)
Yes	41 (63.1%)	9 (52.9%)
Missing	3 (4.4%)	0 (0%)
Average disability score (mean TQS score)		
Mean (SD)	0.234 (0.197)	0.200 (0.169)
Median [Min, Max]	0.200 [0, 0.800]	0.200 [0, 0.600]

Abbrevations used in table: Ten Questions Screen (TQS).

### Objective 2: evaluation and refinement of data collection procedures and outcome

The main question to ask regarding this objective is, ‘How appropriate is the data collection procedure for the intended population and aim of the study?’ The children generally had no problems navigating the digital environment independently when the interview was in progress but almost all of them had some level of support from a parent when logging in for the first time. The children responded to the items and questions verbally, by drawing or inserting icons (e.g., a star) with the annotate function in the shared PowerPoint slides in Zoom. It was noted that drawing lines took effort for some of the children, in which case the icons were preferred. The extent to which children utilized the annotate function varied but most children demonstrated that they could use it in the preparatory memory game at least. Most children answered the questions. However, some children reported difficulty in seeing the pictures on the slide with all the activities when choosing their three most important activities.

The interviewers identified a need for improvement concerning compliance with the interview guidelines, the ability to solve problems during the interview, the preparations, and the allocated time (see Figure 2). Changes were made to the interview guide between the first and second (e.g., adaptations of wording to better fit the digital format) and the sixth and seventh interviews (e.g., the interviewer’s use of the annotate function when giving instructions on the learning tasks was emphasized). The interviewers’ notes concerning the need for improvement included (1) during the interview to repeatedly confirm verbally that the digital environment is working as intended for the child (in one case the child could not see any of the pictures in the slides for several minutes, but did not mention it), (2) to set aside more time (one-hour minimum) per interview in case of technical problems, and (3) to increase knowledge about the Zoom interface on different types of hardware (mobile phone, tablet, and computer).

### Objective 3: evaluation of acceptability and suitability of the study

The main question to address this objective is, ‘Are the study procedures suitable for and acceptable to participants?’ The participants generally adhered to the planned interview procedure. Interviews were completed in 35.3 min on average (range 22–60), excluding the time used for administering the feasibility questionnaire and the short, separately scheduled, preparatory meeting. Even though the children were instructed that they could ask for a pause whenever they felt they needed one, this never occurred. One of the children chose to participate with the camera turned off and by answering exclusively through the annotate function, i.e., with no verbal responses to questions. Child involvement in the interview was generally perceived as high by the interviewer and only two of the participants said that the interview was boring (see Figure 3). There were no serious unexpected adverse events (e.g., signs of discomfort) and few technical issues during the actual PmP interviews. In two cases, short interruptions in the interviews were caused by the child or parent receiving a telephone call on the same device they were using for the PmP interview. The other technical challenges were identified and dealt with during the preparatory meeting. For one family, there was an issue with a microphone malfunctioning, which was solved by replacing it for the actual interview. At least seven children struggled to find the annotate function in Zoom and three of them had to switch devices to get it working (from computer to computer and from computer to smartphone). All parent–child dyads were able to solve the problems that arose somehow but, for the interviewer, it was not always clear exactly what had caused the problem and how it was solved.

### Objective 4: evaluation of resources needed for managing the study

The main questions to ask to address this study objective are ‘Does the research team have the resources and ability to manage the study?’ and ‘What are the ethical implications and necessary considerations of the study?’ The video interviews combined with the preparatory meetings (10–15 min) took somewhat longer than the 30-min approximation of the time needed for the interview mentioned in the PmP manual. Still, there is no reason to believe that a partial transfer to a video-based procedure would increase the time and resources needed for the data collection process as a whole since video-based interviews should lead to less time spent on traveling to data collection sites (participants’ homes, schools, or habilitation services in the CHILD-PMH project). Since data in many projects are collected over large geographical areas, traveling to sites could require a substantial amount of time throughout the projects.

The software applied in the interviews was chosen for its simplicity and familiarity with most academics, and as indicated by the interviews, to many children. Not all children had used Zoom before, but all of them had experience with some form of video communication. The COVID-19 pandemic and the resulting increase in digital meetings have probably contributed to a general increase in relevant skills within this field for many academics. The interviewers in the study were not chosen because of their level of expertise in IT and digital communication. Rather, their skills and experience in the field were in line with academics in general. It is thus unlikely that extensive training would be required to provide data collectors working with PmP or other self-rating instruments in a video format with the fundamental technical skills needed to administer the interview. However, the difficulties in assisting the children with some of the technical issues indicated that some skills and knowledge about the digital environment may be needed to facilitate technical problem-solving during the interview. Thus, one could consider letting data collectors who are more skilled in video communication do all the video-based interviews rather than dividing them among all data collectors in a project. It is also advised that all data collectors that are scheduled for video interviews first try out and practice the procedure, ideally on all possible forms of devices (smartphone, tablet, laptop, etc.) that may be used by participants.

Furthermore, some of the children demonstrated a high degree of familiarity with the digital environment and responded swiftly to the questions, which put further demands on the interviewers’ capability to navigate the digital environment. Apart from the potential effects on costs/savings related to time, partly switching to the described procedure is not expected to lead to additional costs in terms of technical equipment, since both software and hardware are part of the standard equipment of most academics. Of course, video interviewing presupposes that the child has access to a device capable of running the necessary application. In the current study, 13 children (76.5%) used a computer, three (17.6%) a mobile phone, and one (5.9%) a combination of both (due to problems accessing the annotate function on one of the devices).

There are however a few ethical considerations that need to be addressed. For example, moving to a digital environment may lead to new challenges in controlling and protecting confidentiality. This question relates to what sort of information is being collected by the companies providing the video service, but also to who may be listening in on the interview without being visible through the participating child’s web camera. In some of the interviews, a parent’s presence in the room was only indicated by the child’s gaze or when technical issues emerged, and the parent assisted the child with solving them. The presence of a parent may affect how a child responds to certain questions, and if the parent is not visible in the webcam frame, there is a risk that such problems may pass unnoticed.

### Objective 5: preliminary evaluation of the children’s responses

**Figure 4:**
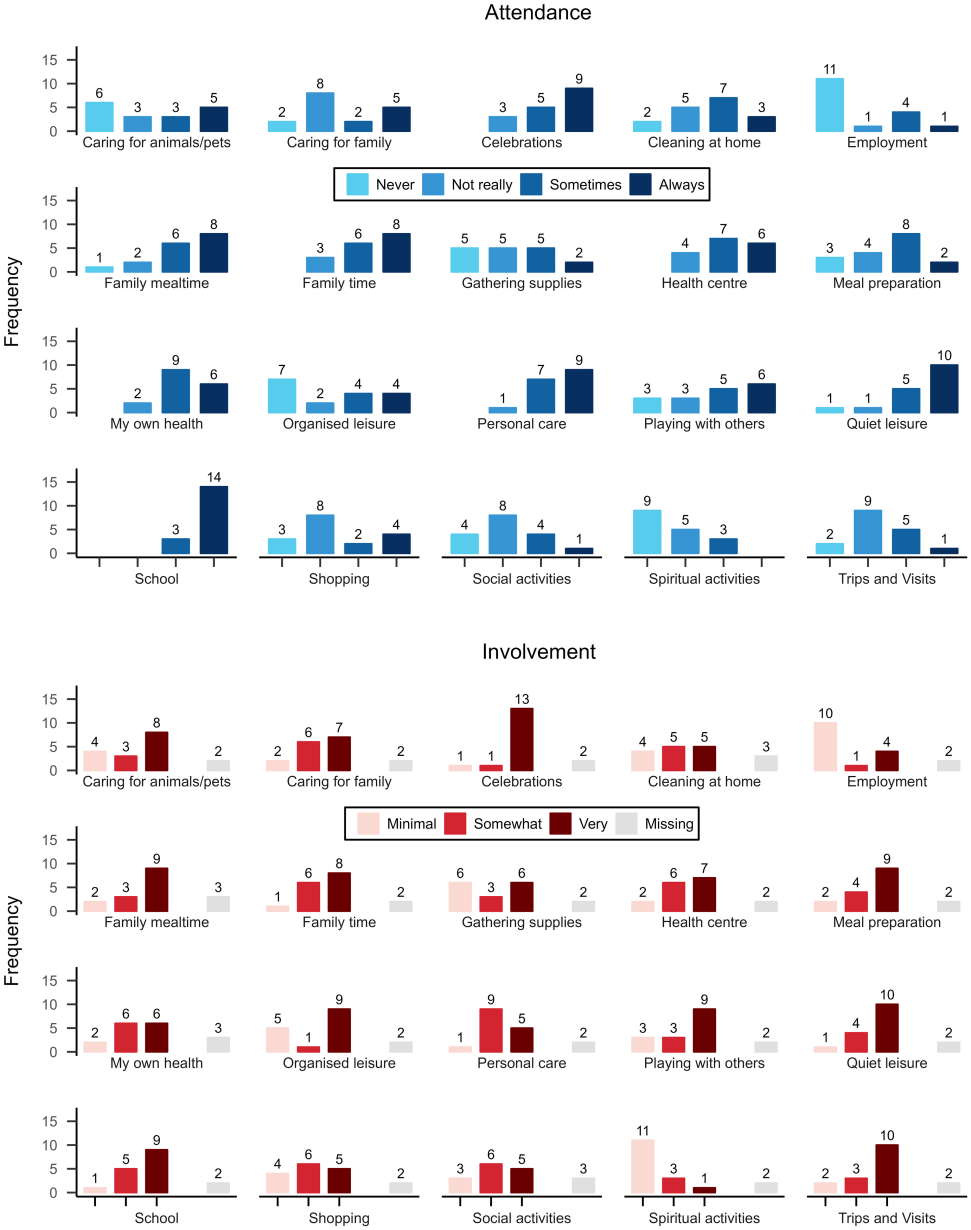
Picture my participation: attendance and involvement in activities.

The main question to address this objective is: ‘Does the study show promise of being successful with the intended population?’ A visual inspection of the PmP responses (see Figure 4) did not reveal any distinct problematic patterns. There was a general skewness toward more positive responses, but all response options were utilized across items. The highest summed ratings were seen in ‘School’ for attendance and ‘Celebrations’ for involvement, and the lowest for ‘Spiritual activities’ (for both). In most activities, high involvement accompanied high attendance and vice versa, but there were a few exceptions, such as ‘Trips and visits’ where response distributions differed. Most difficulties that arose during the interview were related to the PmP instrument rather than the video format *per se*. The amount of missingness was relatively low (6.5%) and originated from four participants’ inability to respond to involvement items. For two of the participants, the interviewer chose not to administer the items from the involvement dimension in PmP, since it was clear that they would be too cognitively demanding for the child. The remaining missing data was derived from two interviews where the participating children found specific questions illogical or not possible to answer correctly. There was no missing data in the attendance subscale. Nine of the children indicated that the questions were hard to understand to some degree (see Figure 3). Primarily, this concerned the barriers and facilitators part of the interview, which demands high cognitive capacity due to its level of abstraction.

## Discussion

In this study, we aimed to investigate the feasibility of administering the self-rating instrument PmP via a video communication tool for children with DD. We chose to use Zoom as a video communication platform and PmP as an example of a self-rating instrument developed to measure children’s participation. By conducting this study, we gained further knowledge in how to use video communication to facilitate children’s self-rating of subjective experiences such as participation in research and/or clinical practice, when situational or geographical conditions may hinder IRL data collection. We learned that guiding self-ratings through video communication may be a feasible option when assessing participation in everyday activities in a non-negligible proportion of children with DD aged 11–14. A considerable share of children approached agreed to participate and went through with the interview. The applied procedure and application were well tolerated by the children and did not lead to problematic levels of attrition or any adverse events. A few technical issues appeared but were generally solved by parents and children before the actual interviews. No special training or expensive equipment was needed to conduct the interviews.

The study touches on the core components of participation identified in the fPRC framework (attendance and involvement; Imms et al., 2016, 2017) of children with DD across three layers: (1) the research process, (2) the digital environment where the assessments were conducted, and (3) the everyday activity domains assessed with PmP. While the study did not investigate the general feasibility of self-rating procedures for children with communicative and cognitive impairments, it aimed to identify specific cognitive and communicative barriers inherent to the digital format. The results revealed that a video-based interview procedure could facilitate attendance in research for some children with DD, most clearly indicated by the participant who chose to answer the questions via the chat function within the video application. This child would have refused participation in an IRL interview. However, relying solely on video interviews when collecting data on participation for children with DD could risk introducing bias in the results, as evidenced by differences in TQS profiles among the children in the study. To reduce this bias, guided self-rating through video communication could be offered as an option, rather than the sole method for data collection. It is important to note that the procedure may be less feasible for children with individualized pictorial support systems. In this study, we relied on the pictorial support included in PmP, which was transferred to the digital format in advance. However, parents of children who require more specific accommodations may have declined participation. It is also worth noting that children with DD such as dyslexia or developmental coordination disorder, with less severe cognitive and communicative impairments, were excluded since participants were recruited through clinics that do not provide services for children with such disabilities. It is reasonable to assume that video interviewing could be a feasible option for an even higher proportion of children with less pervasive diagnoses.

The level of child involvement in the video interviews was high, according to the interviewers. This impression was partially supported by the children rejecting the notion of the interviews as “boring.” Barriers to participation in the interviews were often related to aspects of PmP rather than the digital environment, as demonstrated by some children not comprehending the involvement items. However, the technical problems that occurred during some of the interviews highlighted a feature of the digital environment that may increase implicit demands on expressive language ability. In Zoom and other similar video communication platforms, the environment is only partially shared. The interviewer cannot directly perceive the same things as the child or control all parts of the environment. For example, the Zoom interface differed somewhat between devices, and finding the annotate function was not always straightforward. Further, one child failed to mention that the sharing of PowerPoint slides had stopped working for several minutes. This indicates that problem-solving and participation may be more dependent on the child’s ability to verbally explain what they perceive and to understand instructions on how to navigate the environment in digital interviews. In an IRL interview, it is likely easier for the interviewer to use clues from the environment to identify and solve problems. For children with language impairments, such as those with developmental language disabilities, this aspect of the digital environment is more likely to cause participation restrictions than for children with typical language development. The effect is likely to be larger in less structured interview settings. To reduce this dependency, interviewers should be made aware of reoccurring problems and their typical causes in different digital environments.

Concerning the participation in everyday activities measured by PmP, results need to be interpreted with caution due to the low number of participants in the study. As when administering PmP in person to children with an intellectual disability (Arvidsson et al., 2020, 2021), the responses were positively skewed, which is likely to do with the PmP instrument as such rather than the digital format of the interview. However, in contrast to findings in earlier studies (Arvidsson et al., 2020, 2021), there was no missing data in the attendance subscale.

PmP does not differentiate between activities in a digital and non-digital environment but, notably, the level to which the activities can be performed in a digital context differs. Trips and visits to friends and family are likely to be much more difficult to transfer to a digital environment than quiet leisure, which may involve activities such as playing video games online. For this reason, it is interesting to compare the relatively low rate of attendance in organized leisure in the present study, which probably most often takes place IRL, to the higher rate in quiet leisure. Whether digitalization could play a role in enabling higher rates in certain activity domains than others needs to be further evaluated in future research.

The results of the study call attention to a few specific ethical challenges in projects including video interviewing. Firstly, additional measures are needed to reduce the risk of sensitive data leaking from the project. It may, for example, be necessary to communicate information that could be used to identify participants (e.g., social security number) separately from the video interview if the information is somehow transferred and/or stored in the hands of a third party or corporation. Secondly, measures need to be taken to get a picture of who is listening to the interview (e.g., family members off-screen). Equally important is giving the participating child a clear picture of the immediate surroundings of the interviewer.

Previous research has proved there are many obstacles to overcome for accessing necessary healthcare interventions for children with DD as well as participation in self-ratings and self-assessments of participation and related constructs (Varghese et al., 2015; Adugna et al., 2020; Doherty et al., 2020). Although tentative, the results from this study are promising since the use of video communication could increase the accessibility of research projects including self-ratings of participation in children with DD. It is reasonable to assume that the results would generalize to other outcome measures where the subjective experience is of key importance, such as mental health problems or well-being, as well as to older individuals with similar types and levels of disability. Before being applied in a larger project, it is recommended that the procedure and necessary applications are tested and practiced by all data collectors, on different types of devices. The results are also applicable in rehabilitation and habilitation services where participation is an important outcome, at least in environments where digital solutions are available. In a recent scoping review, professionals and service users reported several benefits of using digital meetings, i.e., teletherapy, as a complementary alternative to IRL meetings with professionals (Benz et al., 2022). Teletherapy was perceived as resourceful, increased the accessibility of service, and contributed to opportunities to connect with others. It could be presumed that children and youth with DD would similarly benefit from teletherapy. One finding from the present study, that may apply to teletherapy as well, is that unexpected events such as technical issues are likely to reveal demands on language abilities. The more familiar the interviewer is with the digital environment in the treatment, the more likely it is that he or she could assist the child, without having to rely on the child’s expressive communicative abilities. In addition, in teletherapy, where the procedure may be less predictable than in the current study, a more flexible pictorial support system is likely to be needed.

## Limitations

The major limitation of the present study is the relatively small sample and the sampling strategy. The communicative and cognitive functioning of the participants were not assessed in the study, contributing to the limitation in the generalizability of the findings. The study does not provide an answer to the question of exactly where to draw the line on which children can and cannot validly respond to, what are assumed to be, cognitively accessible questions on subjective phenomena like participation in a video format. It also does not answer how cognitively accessible the questions and procedure are, or whether the video format as such has an impact on how children respond to questions concerning participation and related constructs. Although further research is needed to answer these questions, there are some indications that self-ratings for children with DD actually can be facilitated through the use of different apps and different forms of video communication (Kaiser et al., 2001; Stephenson and Limbrick, 2015). Also, the results of this study give enough confidence in the feasibility of the approach to encourage future use of video communication to guide self-ratings of participation and related constructs in children with DD.

## Data availability statement

The datasets presented in this article are not readily available because of commitments in the ethical approval. However, anonymized data without participant characteristics is available upon request. Requests to access the datasets should be directed to magnus.ivarsson@liu.se.

## Ethics statement

The studies involving human participants were reviewed and approved by the Swedish Ethical Review Authority (case number 2019-05028). Written informed consent to participate in this study was provided by the participants’ legal guardian/next of kin. The individual(s) provided their written informed consent for the publication of any identifiable images or data presented in this article.

## Author contributions

MI and AA: conceptualization, data curation, formal analysis, investigation, methodology, project administration, software, visualization, writing—original draft, writing—review and editing. LA: conceptualization, data curation, formal analysis, methodology, project administration, visualization, writing—original draft, writing—review and editing. All authors contributed to the article and approved the submitted version.

## Funding

This work was supported by the Swedish Research Council under Grant 2018-05824_VR.

## Conflict of interest

The authors declare that the research was conducted in the absence of any commercial or financial relationships that could be construed as a potential conflict of interest.

## Publisher’s note

All claims expressed in this article are solely those of the authors and do not necessarily represent those of their affiliated organizations, or those of the publisher, the editors and the reviewers. Any product that may be evaluated in this article, or claim that may be made by its manufacturer, is not guaranteed or endorsed by the publisher.
