# Supplementary material for: Self-rating via video communication in children with disability – a feasibility study
Source: Front Psychol. 2023 May 2;14:1130675. doi: 10.3389/fpsyg.2023.1130675 (PMC10187543; doi:10.3389/fpsyg.2023.1130675)
Supplement: Supplementary file 1 [file Data_Sheet_1.pdf]

Supplementary material: Self-rating via video communication in children with disability – a feasibility study

Supplementary Figure 1

Example of how the Picture my Participation questions about attendance in activities (in this case "Cleaning at home") was displayed as a slide in PowerPoint. The response options are "Always" ("Alltid"), "Sometimes" ("Ibland"), "Not really" ("Sällan"), and "Never" ("Aldrig")

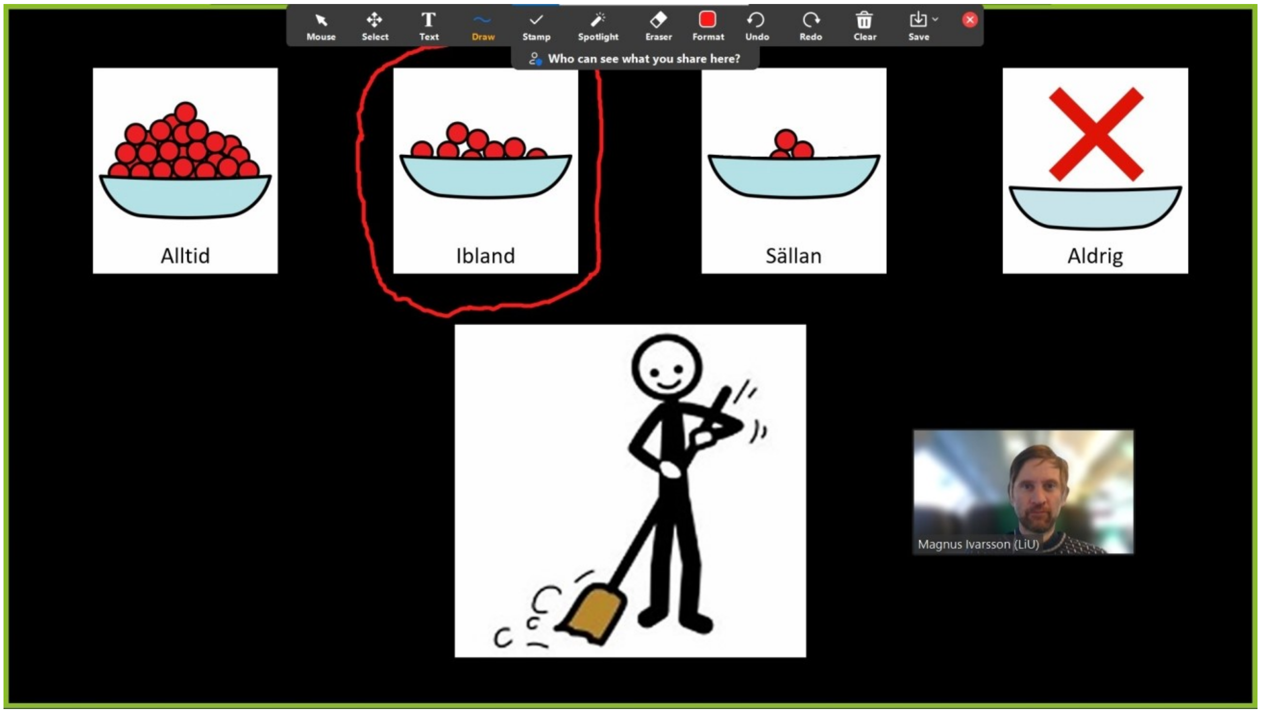

Note. The pictographic symbols used are the property of the Government of Aragón and have been created by Sergio Palao for ARASAAC (available at https://arasaac.org/). They are adapted/reproduced with permission from the Government of Aragón. Some of the pictures used during the actual interviews could not be displayed for copyright reasons and were replaced with equivalent pictures from the ARASAAC series. A few of the ARASAAC pictures have been modified to fit the text in Picture my Participation.

Supplementary Figure 2

Example of how the Picture my Participation questions about involvement in activities (in this case "Cleaning at home") was displayed as a slide in PowerPoint. The Response Options Are "Very" ("Mycket"), "Somewhat" ("Lite"), and "Not" ("Inte alls")

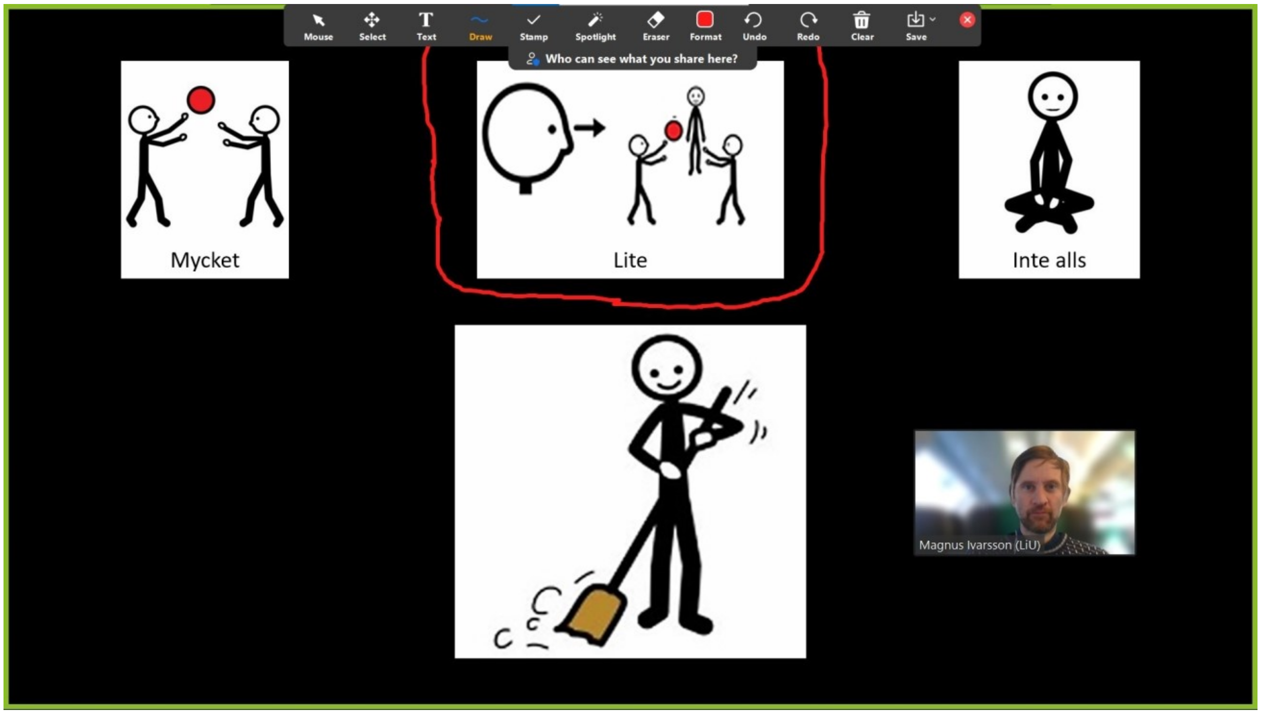

Note. The pictographic symbols used are the property of the Government of Aragón and have been created by Sergio Palao for ARASAAC (available at https://arasaac.org/). They are adapted/reproduced with permission from the Government of Aragón. Some of the pictures used during the actual interviews could not be displayed for copyright reasons and were replaced with equivalent pictures from the ARASAAC series. A few of the ARASAAC pictures have been modified to fit the questions in Picture my Participation.
